# Bioactivity of silverleaf nightshade (*Solanum elaeagnifolium* Cav.) berries parts against *Galleria mellonella* and *Erwinia carotovora* and LC-MS chemical profile of its potential extract

**DOI:** 10.1038/s41598-024-68961-z

**Published:** 2024-08-13

**Authors:** Abdel Nasser A. Kobisi, Mohamed A. Balah, Ahmed R. Hassan

**Affiliations:** 1 Plants Protection Department, Desert Research Center, Cairo, Egypt; 2 Medicinal and Aromatic Plants Department, Desert Research Center, Cairo, Egypt

**Keywords:** *Solanum elaeagnifolium*, *Galleria mellonella*, *Erwinia carotovora*, Insecticidal activity, Bioassay-guided approach, LC-MS, Biological techniques, Plant sciences

## Abstract

Natural products received much attention as an environmentally beneficial solution for pest management. Therefore, the extracts of invasive silverleaf nightshade (*Solanum elaeagnifolium* Cav.) weeds using their berries parts (seeds, peels and mucilage) supported by bioassay-guided fractionation were tested against both the greater wax moth (*Galleria mellonella*) and *Erwinia carotovora* pv. *carotovora* causes of the blackleg of potatoes*.* The seeds and peels of *S. elaeagnifolium* were successively extracted by maceration using dichloromethane (DCM), ethyl acetate (EtOAc), and ethanol (EtOH), respectively. While, its mucilage was extracted using EtOAc. The successive EtOH extract of the plant seeds had promising inhibition efficacy and the best minimal inhibition concentration (MIC) of 50 µg/ml against *E. Carotovora* amongst other extracts (DCM and EtOAc of the plant berries parts). Depending on dose response activity, EtOH extract had *G. mellonella* larval mortality and pupal duration rates (LC_50_; 198.30 and LC_95_; 1294.73 µg/ml), respectively. Additionally, this EtOH extract of seeds was fractionated using preparative TLC to three characteristic bands. The insecticidal and bacterial activities of these isolated bands (SEA, SEB, and SEC) were evaluated at a dose of 100 µg/ml, causing mortality by 48.48, 62.63 and 92.93% (*G. mellonella* larvae) and inhibition by 15.22, 0.00 and 31.66 mm (*E. carotovora*), respectively. Moreover, the separated major three bands were tentatively identified using LC-ESI-MS analysis revealing the presence of two phenolic acids; chlorogenic acid (SEA) and dicaffeoyl quinic acid (SEB) in addition to one steroidal saponin (SEC) annotated as borassoside E or yamoscin. Finally, the plant seeds’ successive EtOH extract as well as its active constituents, exhibited potential broad-spectrum activity and the ability to participate in future pest management initiatives. A field study is also recommended to validate its bio-efficacy against selected pests and to develop its formulations.

## Introduction

Crop productivity largely depended on inputs like inorganic fertilizers, synthetic pesticides, and genetically modified organisms to meet the demand for food^1,2^. These pesticides have been used carelessly, which has resulted in undesirable effects such as environmental degradation, harm to non-target creatures, pesticide residues polluting food and feed, insect resurgence, genetic variety in plants, and detrimental effects on biodiversity^3,4^. The harmful effects of improper and excessive use of chemical pesticides made it necessary to find alternate methods of pest control^5^. Thus, there is a stronger emphasis on the creation of botanical pesticides^6^. Various studies have been conducted on the insecticidal potential of some plant extracts^7,8^. These plants can contain important insecticide molecules for the control of pests. Weeds’ potential as a source of bio-insecticides is expected to elevate them from a plant with no economic value to a useful plant^9^. Thus, the identification of weeds with insecticidal activity through testing their toxicity to insects should be investigated^10^.

The genus *Solanum* is known to contain several secondary metabolites in its leaves, fruits, and roots, such as alkaloids^11^, flavonoids^12–14^, and terpenes^15^, providing it with a strong allelopathic effect^16,17^. These plant-derived natural compounds called allelochemicals^18^, which affect insects of all orders, particularly herbivorous insects and other pests, such as molluscicides, nematicides, and bactericides^19,20^, acaricides and fungicides^21^. *Solanum elaeagnifolium* Cav. (Solanaceae), known as Silver-leaf nightshade and originating from the United States, is one of the worst invasive alien plants in the Mediterranean Basin^22,23^. It has potent insecticidal and repellent potential against plant pests that are supplied as an alternative pesticide for crops^24^, and antimicrobial activity^25^. Water-soluble extracts of silver-leaf nightshade foliage inhibited the germination and root growth of cotton and lettuce, respectively^26^. We can sum up some of the following *S. elaeagnifolium*-based compounds by reviewing the literature. For instance, solasodine (a steroidal alkaloid) is abundant in the Solanaceae family^27,28^. Cholesterol, campesterol, sitosterol, stigmasterol, Δ^5^-avenasterol, Δ^7^-avenasterol, Δ^7^-stigmastenol and *α*-spinasterol were identified from the seeds of 13 species of *Solanum*, including silverleaf nightshade^29^. Glycoalkaloids were isolated from silverleaf nightshade seeds and leaves^30^. Kaempferol-3-O-*β*-D-(6′′'-O-*cis*-cinnamoylglucoside, kaempferol and kaempferol-3-glucoside were characterized from aerial parts of silverleaf nightshade^12^. *β*-sitosterol-3-O-*β*–D-glucoside, quercetin-3-O-*β*-D-glucopyranoside, *β*-sitosterol, stigmasterol, and kaempferol were isolated from EtOAc and 25% methanol/ EtOAc fractions of S. elaeagnifolium^14^. A flavonoid C-glycoside (kaempferol 8-C-*β*-galactoside) was identified from the aqueous methanolic plant extract^31^. 2-(2-hydroxyphenoxy)-3,6,8-trihydroxy-4H-chromen-4-one, quercetin, rutin and mangiferin were isolated from the plant^32^. 2*R*,3*R*-5,7,4′-trihydroxy–dihydroflavon-3-O-*α*-D-glucopyranosyl-6′′-O-*β*-D-glucopyranoside -6′′-*p*-hydroxy benzoate and kaempferol-3-(6′-coumaroyl glucoside) were also isolated from this plant^33^. Moreover, the previously isolated Monoacylated flavonoid glucoside (kaempferol-*β*-D-(6′′-O-*cis*-cinnamoyl glucoside) and mangiferin along with chlorogenic acid, kaempferol, coumaroyl glucoside, coumaroyl quinic acid and dicaffeoyl quinic acid were the most active constituents in the plant seeds against some weeds^13^.

The greater wax moth *Galleria mellonella* is a ubiquitous pest of honeybee colonies globally causing damage to wax combs in stressed beehives and stored beekeeping equipment, where the larvae feed and transform into moths^34^. It is increasingly used in scientific research as a model for the investigation of insect biochemical and molecular levels^35^ and as an alternative non-mammalian model for the study of fungal virulence and pathogenesis^36^. It is expected that the species will continue to spread to unmanaged areas, which may be associated with changing climatic conditions^37^. On the other side, *Erwinia carotovora* is one of the destructive pathogens of postharvest vegetables worldwide^38^, especially potatoes (*S. tuberosum*), green peppers (*Capsicum annuum*), and Chinese cabbages (*Brassica campestris *subsp. pekinensis)^39^. It causes soft rot disease of economically important crops, such as tomatoes, broccoli, and cucumbers^40^. This pathogen causes soft rot of stem and tubers before and after harvest and greatly reduces yields of potatoes^41^.

To determine whether there are any natural alternatives to synthetic pesticides, *S. elaeagnifolium* was selected from the genus *Solanum* which widely invaded in the western and eastern Mediterranean coast regions of Egypt. It has been interfered with negatively by allelopathic capabilities and inhibited the native species growth. Therefore it is nominated as the source to obtain safe natural pesticides that may be used to protect cropping systems. We hypothesize that *S. elaeagnifolium* as the most spreading invasive weed in Egypt recently has high bioactive constituents potentials that may help in pest control and can be developed as natural pesticides and may be used in the future in integrated pest control strategies. Based on this background, the study aimed to investigate invasive *S. elaegnifolium* berries (seed, peel, and mucilage) insecticidal and bactericidal properties. The bioactive components isolation and characterization were performed, and the relationships between constituents’ activity and pest response were quantified.

## Material and methods

### Plant materials

The fruit berries of *Solanum elaeagnifolium* Cav. were collected from August to November 2021 from Al-hammam and Borg Al-arab regions, Egypt. All the steps from plant collection and experimentation on wild *S. elaeagnifolium* plants are in compliance with relevant Institutional, National, and International guidelines and in accordance with local legislation and with permissions from Desert Research Center. The collected plant was authenticated by Dr. Ghaly, Desert Research Center, Cairo, Egypt. A voucher sample (CAIH-30-11-2021-S) has been deposited at the Herbarium of DRC, Cairo, Egypt. After collection, part of the fruit berries was shredded to obtain the peels (dried and ground to fine powders), the mucilage obtained from berries around the seeds by washing in distilled water (DW), and the seeds (obtained after washing in DW, drying and ground to fine particles). The drying process was implemented at 50 ℃ in an oven for 5 days.

### Extraction and fractionation of *S. eleaegnifolium* (fruit peels, seeds and mucilage)

200 g powder of *S. elaeagnifolium* seeds and peels were successively extracted by maceration using dichloromethane (DCM), ethyl acetate (EtOAc) and ethanol (EtOH), respectively. These three extracts were subjected to a biological screening (in vitro bactericides assay). Mucilage (locular tissue) was removed manually from 200 g of berries by washing in 1L of distilled water using shaking overnight at room temperature. The obtained extract was partitioned with an equal volume of EtOAc three times. The EtOAc extract was dried with a rotary evaporator under a vacuum at 40 ℃. The residues were weighed and kept at − 20 ℃ until used^17^. The most active extract (EtOH extract of seeds) was subjected to further chromatographic fractionation to identify its active phytochemicals. Subsequently, the successive EtOH extract of seeds was initially monitored by analytical normal phase-thin layer chromatography plates (silica gel 60 GF_254_ NP-TLC) using EtOAc—methanol (MeOH)—H_2_O with a percentage of (20: 5: 4). Then, the plate was visualized under UV lamp (254 nm) and immersed in 5% H_2_SO_4_ in MeOH followed by heating at 105 ℃ for 3 min to give three major characteristic spots^42^. Accordingly, about 100 mg of the previously mentioned extract was fractionated using normal phase-preparative-thin layer chromatography (NP-PTLC) on silica gel 70 FM TLC glass plates using the previously used eluent. Consequently, these yielded three major bands or layers. These promising bands were scratched out from the NP-PTLC plates and dissolved in MeOH to afford three isolated fractions coded as SEA, SEB and SEC to evaluate their in vitro insecticidal and bactericidal activities. Finally, these resulting fractions were subjected to LC-ESI-MS/MS to identify their major chemical profile.

### In vitro bactericidal activity

The agar well diffusion method as displayed by Daoud et al*.*^43^ used to screen the antibacterial activities. Molten-cooled Muller Hinton agar (MHA) was used for *Erwinia carotovora* growth and subsequently poured into the Petri dish and one ml of fresh bacterial culture was pipetted in the center and mixed well. The Petri dishes were left until the media cooled and solidified, and well were made by using a Cork Borer (diameter 6 mm). Then, 100 µl from each extract concentration (50, 100, 200, 400, 800 µg/ml) was added to respective wells. DMSO was used as a control treatment. Then, the plates were incubated at 37 ℃ for 18 h. Antimicrobial activity was detected by measuring the zone of inhibition (including the well diameter) that appeared after the incubation period^44^. To determine the minimum inhibitory concentration of mucilage, peels and seed extracts of *S. elaeagnifolium* and the three fractions of EtOH seed extract (SEA, SEB and SEC) against *E. carotovora.* One-fold dilution was used to prepare a concentration of 800 µg/ml of mucilage, peels, and seed extracts, while the three fractions (SEA, SEB, and SEC) were prepared from 8, 16, 32, 64, and 128 µg/ml. Then, three wells were made on each containing nutrient agar petri dishes, and 100 µl of each concentration was transferred to the respective wells. Plates were kept in the refrigerator for 30 min and then incubated at 37 ℃ for 24 h. The MIC was considered to be the lowest concentration that inhibited the growth of the respective microorganisms. All assays were performed in triplicate and DMSO was used as a control.

### In vitro insecticidal activity

#### Insect rearing

*Galleria mellonella* (Lepidopetra: Galleridae) was obtained from the Entomology Unit of the Plant Protection Department, Desert Research Center, Egypt. This insect was reared in accordance with Metwally et al.^45^. For adults emerging and egg-laying, *G. mellonella* pupae were placed in a glass container (15 cm in diameter and 25 cm in height), covered with plain paper, and provided with paper cone with lids attached to the outside of the glass container. Eggs were laid at the base of the paper cones, which were continuously removed for egg collection and replaced by new ones. *G. Mellonella* eggs were placed in a glass container (about 3 litters in size) containing 3–4 cm of a semi-synthetic diet (wheat flour 350 g, corn flour 200 g, milk 130 g, backing yeast powder 70 g, honey 100 ml, glycerin 150 ml). The containers were covered with plain paper and fitted in place with two rubber bands. Rearing containers were incubated at 28–30 ℃ with 60–70% RH. More diet was added to the developing larvae as needed until pupation.

The feeding toxicity of the crude extracts of the various fruit sections of *S. elaeagnifolium* (peels, mucilage and seeds) was evaluated against larvae of *G. mellonella*. Firstly, an artificial diet was prepared and weighed (10 g) to be transferred into each transparent cup (61 × 73 cm) to be combined with 5 ml of *S. elaegnifolium* crude extracts. The extract (1000 µg/ml) and diet were then vigorously swirled to ensure a consistent mixing of the two components. To maintain consistency among artificial diets, the diet was given three hours to dry in an aseptic atmosphere. The diet was simultaneously treated with distilled water, which served as a control (negative control). Five healthy larvae (active), uniformly asymmetrical (1st instar), and pre-starved (2 h) were added to one cup. Each treatment was replicated ten times. Larval mortality was taken every two days for 25 days. The experiment was conducted at 28 ± 2 ℃ and 70 ± 2% temperature and relative humidity, respectively^46^. The larvae were left until they entered the pupal stage in order to observe the effect of the crude extracts of the various fruit sections of *S. elaeagnifolium* on the pupae.

To find out the lethal concentration of each part of fruit extract (peels, mucilage, and seed), a range-finding test was conducted. For this, initially, a 600 μg/ml concentration of fruit part extracts was tested and observed to cause larval mortality. Further, five lethal concentrations were estimated for (100, 200, 300, 400, 500 μg/ml) extracts against *G*. *mellonella* larvae that were adjusted until targeted 20–95% larval mortality (10 g of artificial diet combined with 5 ml of each concentration were exposed to the air to dry for three hours). Each treatment was replicated ten times, with five larvae per replication. Larval mortality was taken on every two days (48 h) for 25 days to calculate final larval mortality^44^. Data of larval mortality are shown as corrected mortality according to Abbott^47^; (% Responded in treatment—% responded in control) / (100—% responded in control) × 100. To estimate the lethal concentrations (LC_50_, LC_90_) probit analysis was carried out according to Akçay^48^.

As mentioned above in the method of Dose-response toxicity, the same steps were carried out with three isolated fractions (SEA, SEB, and SEC) resulting from the fractionation of EtOH extract of the seeds at concentrations of 25, 50, 75, and 100 (μg/ml).

### LC-ESI-MS/MS analysis

The chemical constituents of the fractionated bands (SEA, SEB and SEC) obtained from the successive EtOH extract of seeds were identified by LC-ESI-MS instrument using an Acquity-UPLC™ system (Waters, Milford, USA) equipped with a BEH-C18 column, with 2.1 × 50 mm dimensions and a 1.7 μm particle size. The mobile phase consists of H_2_O containing 0.1% formic acid (A) and MeOH acidified with 0.1% formic acid (B) with a flow rate of 0.2 ml min^−1^ in a gradient method as follows: 0–2 min (90% of solvent A), 2–5 min (70% A), 5–15 min (30% A), 15–22 min (20% A), 22–26 min (20% A), 26–29 min (100% B), 29–32 min (90% A)^49^. Mass spectra were recorded using XEVO TQD triple quadrupole mass spectrometer equipped with ESI detector in both negative and positive ion modes within the *m/z* range of 100 and 1000. The following parameters of LC/MS analysis; source temperature 150 ℃, dissolution temperature 400 ℃, cone voltage 30 eV, capillary voltage 3 kV, cone gas flow 50 L/h, and dissolution gas flow 600 L/h were adjusted. Mass spectra were processed using the Masslynx 4.1 software and the compounds were annotated by comparing their molecular ion peaks and fragmentation patterns with the reported data.

### Statistical analysis

The bioassay data were presented as means ± standard error (SE), and their replications were statistically analyzed by One-way ANOVA to observe the significant differences (*p* ≤ 0.05) between treatments means through Tukey’s HSD post hoc test using IPM SPSS software version 21.

## Results

### Primary screening of seeds, mucilage and peels of *S*. *elaegnifolium* against *E. carotovora*

*S. elaegnifolium* fruit parts of seeds and berry peels were subsequently successively extracted and evaluated against *E. Carotovora* at doses of 50, 100, 200, 400 and 800 μg ml^-1^ concentrations as indicated in Table 1. EtOH was the highest inhibitor extract than DCM, EtOAc with minimum inhibition zones 9.33 and 8.00 mm for seeds and peel parts, respectively. Seeds extract (F = 14.706 P ≥ 0.00) showed the maximum significant inhibitory activity against *E. Carotovora* than fruit peels and mucilage*.*

**Table 1:** The diameter (mm) of the inhibitory zone caused by *Solanum elaeagnifolium* seeds, peels, and mucilage extracts on *Erwinia carotovora*.

Fruit berries	Extract (solvents)	Concentration (μg/ml)						MIC (μg/ml)		F (p _value_)	
		Control	50	100	200	400	800		Extract solvent		Fruit parts
Seeds	DCM	ND	0.00 ± 0.00	7.33 ± 0.47	13.33 ± 0.47	17.67 ± 0.94	21.33 ± 0.47	100	125.5(0.00)		14.706 (0.00)
	EtOAc	ND	0.00 ± 0.00	7.67 ± 0.47	14.67 ± 0.94	19.33 ± 1.25	23.33 ± 1.25	100	277.3 (0..00)		
	EtOH	ND	9.33 ± 1.25	16.33 ± 0.47	20.33 ± 1.25	25.00 ± 0.82	29.67 ± 0.94	50	285.4 (0.00		
Peels	DCM	ND	0.00 ± 0.00	6.00 ± 0.82	12.00 ± 0.82	16.33 ± 1.25	19.67 ± 0.47	100	68.5(0.00)		5.51 (0.02)
	EtOAc	ND	0.00 ± 0.00	6.67 ± 0.47	14.67 ± 1.25	19.00 ± 0.82	22.33 ± 1.25	100	178.5 (0..00)		
	EtOH	ND	8.00 ± 0.82	15.00 ± 0.82	19.00 ± 0.82	23.67 ± 0.47	28.33 ± 0.47	50	235.6 (0.00		
Mucilage	EtOAc	ND	0.00 ± 0.00	6.67 ± 0.47	12.67 ± 0.47	17.67 ± 1.70	22.33 ± 0.25	100	127.86(0.00)		3.78 (0.012)

### The activity of *S*. *elaegnifolium* parts extracts against *G. mellonella* larvae

Based on the bacterial activity, EtOH extract of seed and peels was the most active extract. These extracts and EtOAc extract of mucilage were subjected to insecticidal activity against *G. mellonella* at a dosage of 1000 µg/ml. These extracts had a significant effect on the duration of the larval stage, which was measured at 19.07, 20.27, and 21.41 days, and the pupal stage, which was measured at 9.67, 11.07, and 13.07 days, respectively. Our results were compared with the control treatment, which had larval life spans of 17.74 days and pupal life spans of 8.87 days (Table 2).

**Table 2:** Effect of seeds, peels, and mucilage extracts on larval and pupal duration of *Galleria mellonella* (Mean ± SE).

Extracts	Larval duration (days)	Pupal duration (days)
Seeds (EtOH)	21.40^a^ ± 0.27	13.07^a^ ± 0.25
Barriers peels (EtOH)	20.27^b^ ± 0.16	11.07^b^ ± 0.25
Mucilage (EtOAc)	19.07^c^ ± 0.25	9.67^c^ ± 0.28
Control	17.74^d^ ± 0.07	8.87^d^ ± 0.08
F, (p value)	135.75, (0.000)	73.93 (0.00)

### Lethal concentration of seed, peel and mucilage extracts on *G. mellonella* larvae

In the preliminary test, the EtOH crude extracts of seeds and peels as well as EtOAc mucilage extract of *S. elaeagnifolium* were dose-dependent (100, 200, 300, 400 and 500 µg/ml) on larval mortality percent as shown in Table 3. The highest mortality achieved with 500 µg/ml concentration was 89.08% of seed extract, 40.82% of peel extract and 34.69% of mucilage extract at the same concentration. The EtOH extract of seeds caused the highest toxicity (LC_50_; 198.30 µg/ml and LC_90_; 1294.73 µg/ml) as compared to other tested extracts. According to the LC_50_ of all three extracts, there was an increase in mortality percentage per day with concentrations achieved from all extracts, and EtOH seed extract recorded the highest mortality percentage per day when compared with two other extracts of peels and mucilage. A significant difference between the three extracts in lethal time compared to the control. Whereas, the EtOH extract of seeds was significantly more effective in prolonging the period of larvae and pupae among the three extracts, with a period of 21.40 and 13.07 days, respectively.

**Table 3:** Mortality percentage and lethal concentration values of peels, seeds and mucilage extracts *Galleria mellonella* larvae.

Conc. (µg/ml)	Seeds (EtOH)	Peels (EtOH)	Mucilage (EtOAc)
0	0.00f. ± 0.07	0.00f. ± 0.07	0.00^e^ ± 0.09
100	28.57^e^ ± 0.32	20.41^e^ ± 0.24	12.24^e^ ± 0.37
200	48.98^d^ ± 0.55	24.49^d^ ± 0.49	18.37^d^ ± 0.63
300	61.22^c^ ± 0.68	28.57^c^ ± 0.32	22.45^c^ ± 0.37
400	75.5^b^ ± 0.49	36.73^b^ ± 0.40	28.57^b^ ± 0.63
500	89.80^a^ ± 0.63	40.82^a^ ± 0.51	34.69^a^ ± 0.37
F, (p value)	114.98, (0.00)	36.64 (0.00)	24.34(0.00)
LC_50_(µg/ml)	198.30	1701.95	1410.79
LC_90_ (µg/ml)	1294.73	20840.77	212649.95
R.e. (*Y* = *a* + *b*X)*	y = 0.029x + 3.54	y = 0.033x + 3.454	y = 0.028x + 3.594
R^2^ =	0.990	0.991	0.998

*The slope ± SE of the toxicity vs. concentration curve, and the Chi-Square as accuracy of data fitting to probit analysis in POLO-PlusV2.

### The activity of the three major fractions resulted from the EtOH seed extract against ***G. mellonella ***larvae and ***E. carotovora***

Based on the promising lethal concentration of *S. elaeagnifolium* seed EtOH extract, this extract was further fractionated chromatographically using NP-PTLC to give three major bands or fractions (SEA, SEB and SEC). According to the results in Table 4, the three fractions showed a gradual increase in mortality rates with increasing concentration. From the toxicological perspective, the most potent fraction was SEC by 45.65 and 155.20 µg/ml (LC_50_ and LC_90_) respectively. SEB fraction came in the second rank by its calculated LC_50_ and LC_90_ presenting clearly how the larval death rate rises in direct proportion to the increase in concentrations throughout the days.

**Table 4:** Lethal concentration (LC_50_ and LC_90_ values) of fractionation of *Solanum elaeagnifolium* seeds EtOH extract on larvae of *Galleria mellonella.*

Conc. (µg/ml)	SEA	SEB	SEC
0	0.00^e^ ± 0.01	0.00^e^ ± 0.03	0.00^e^ ± 0.05
25	4.04^d^ ± 0.22	14.14^d^ ± 0.31	28.28^d^ ± 0.29
50	14.14^c^ ± 0.22	26.26^c^ ± 0.33	42.42^c^ ± 30
75	24.24^b^ ± 0.33	36.36^b^ ± 0.13	58.59^b^ ± 51
100	48.48^a^ ± 0.37	62.63^a^ ± 0.37	92.93^a^ ± 22
F (p value)	31.23 (0.00)	127.61 (0.00)	243.14 (0.00)
LC_50_ (µg/ml)	117.66	87.23	45.65
LC_90_ (µg/ml)	479.00	507.91	155.26
R2	0.952	0.993	0.990
R. e. (y = a + bx)	y = 0.036x + 3.283	y = 0.028x + 3.582	y = 0.032x + 3.434

*The slope ± SE of the toxicity vs. concentration curve, and the Chi-Square as the accuracy of data fitting to probit analysis in POLO-PlusV2.

By the twofold dilution method, concentrations that ranged from 8 to 128 µg/ml were tested against *E. carotovora* to determine the MIC of three separated fractions (SEA, SEB and SEC) from EtOH extract of the plant seeds (Table 5). The results confirmed that one of them (SEC) showed the maximum antibacterial activity which increased by concentration rise with an inhibition zone ranging from 10.67 ± 0.72 mm. to 32.00 ± 0.47 mm. This study validates earlier findings in the literature that show an increase in extract concentration (%) directly correlates with an increase in antibacterial activity. The second fraction (SEA) had less antibacterial activity when compared with (SEC) at the same concentration, while the third fraction (SEB) has no antibacterial activity. By looking at the results of MIC, the SEC fraction had the best one with 16 µg/ml while the SEA band was 64 µg/ml).

**Table 5:** Minimal inhibitory concentration (MIC) of bands (SEA and SEC) of EtOH extract of *Solanum elaeagnifolium* seeds with mean ± SE.

Conc. µg/ml	SEA	SEC
8	ND	ND
16	ND	10.67 ± 0.72
32	ND	18.33 ± 0.54
64	8.33 ± 0.72	26.33 ± 0.27
128	13.67 ± 0.98	32.00 ± 0.47
MIC	64 µg/ml	16 µg/ml
F (p value)	12.54 (0.00)	28.76 (0.00)

*ND* not detected.

**Figure 1:**
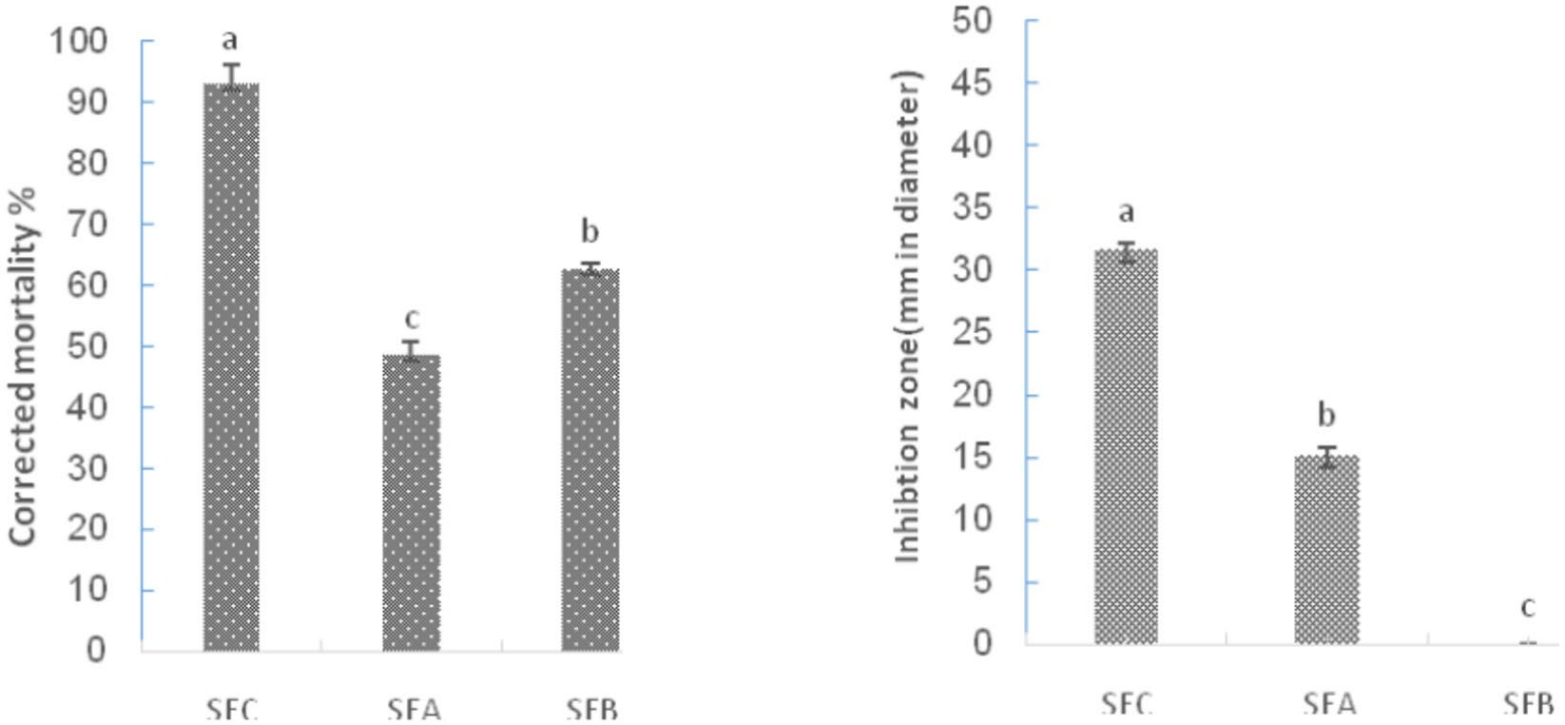
Activity of SEA, SEB and SEC against *Galleria mellonella* larvae and *Erwinia carotovora* at the dose of 100 µg/ml.

As we have mentioned above, the successive EtOH extract of *S. elaeagnifolium* seeds which has the highest potential activity subjected to more fractionation on preparative NP-TLC plates using EtOAc–MeOH–H_2_O (20: 5: 4). This chromatographic process revealed three characterized bands or fractions as manifest by the presence of two bands (coded as SEA and SEB) showed quenching under UV at 254 nm and one band (coded as SEC) charred when sprayed with sulphuric acid (5% in MeOH) accompanied by heating at 105 °C for 3 min. The insecticidal activity of SEA, SEB, and SEC against *G. mellonella* larvae (F = 47.77, *p* < 0.00) showed 48.48, 62.63 and 92.93% (corrected mortality) at doses of 100 µg/ml. While their bactericidal activity against *E. carotovora* (F = 775.9, *p* < 0.00) was 15.22, 0.00, and 31.66 mm (inhibition zone), respectively, relative to its respective control (Fig. 1).

### LC-ESI-MS/MS analysis of ***S. elaegnifolium*** seeds EtOH extract and its three major bands (SEA, SEB and SEC)

**Figure 2:**
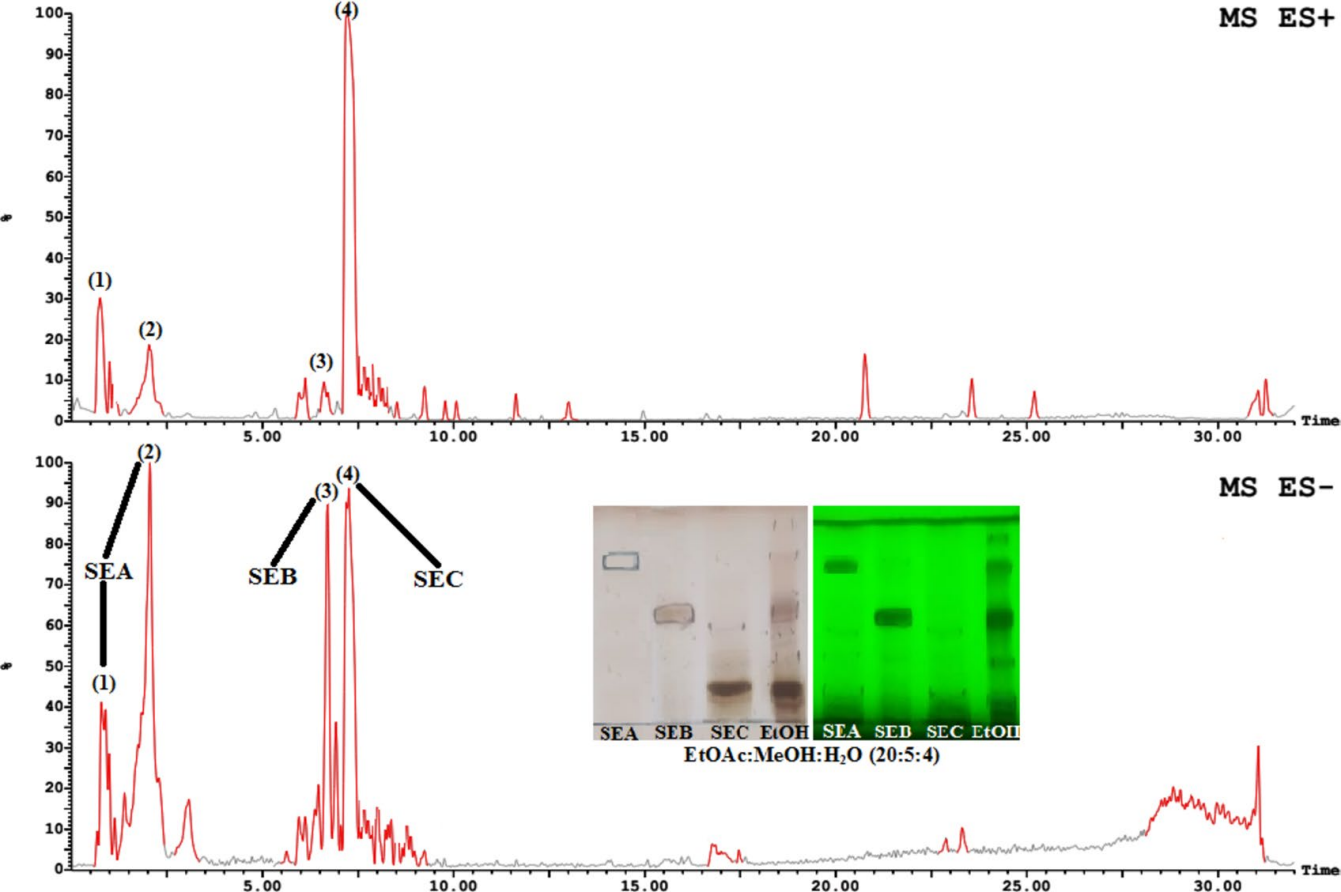
LC-ESI-MS base peak chromatogram of *Solanum elaeagnifolium* EtOH seed extract and its active fractionated bands.

**Figure 3:**
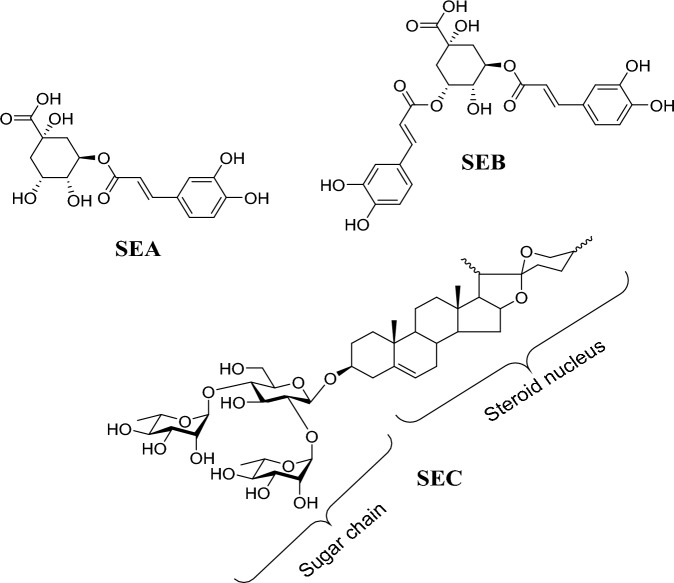
Chemical structures of identified active compounds from *Solanum elaeagnifolium* EtOH seed extract.

The chemical components of the three isolated bioactive bands have been identified using LC-ESI-MS/MS. Its base peak chromatogram in both ionization modes (positive and negative ion modes) is shown in Fig. 2. Identification of the active constituents was based on the mass spectra (molecular ion and fragmentation patterns) and retention time (R_*t*_) of each annotated compound. The assigned active compounds and their fragmentations were summarized in Table 6 and the chemical structures were illustrated in Fig. 3. The R_*t*_ of three bands was found at 0.78, 2.02 for SEA, 6.71 for SEB and 7.21 min for SEC, respectively.

**Table 6:** The major phytochemicals of the successive EtOH extract of *Solanum elaeagnifolium* seeds using LC–ESI–MS/MS.

No	R_t_ (min)	Positive mode *(m/z*)	Negative mode *(m/z)*	Other fragment ions *(m/z)*	Identified compound
1&2	0.78&2.02	377.13 [M + Na]^+^, 355.13 [M + H]^+^& 377.12 [M + Na]^+^, 355.13 [M + H]^+^	707.26 [2 M − H]^−^, 353.15 [M − H]^−^& 707.28 [2 M − H]^−^, 353.15 [M − H]^−^	163^a^[M + H − 192]^+^ 191^b^[M − H − 162]^−^& 163^a^[M + H − 192]^+^ 191^b^[M − H − 162]^−^	Chlorogenic acid (SEA)
3	6.71	539.21 [M + Na]^+^, 517.22 [M + H]^+^	515.17 [M − H]^−^	355^a^ [M + H − 162]^+^, 163^a^[M + H − 192]^+^, 353^b^[M − H − 162]^−^	3,5-Dicaffeoyl quinic acid (SEB)
4	7.21	869.37 [M + H]^+^	912.68 [M + HCOO]^−^	723^a^[M + H − 146]^+^, 576^a^[M + H − 146 − 146]^+^, 414^a^[M + H − 146 − 146 − 162]^+^	Borassoside E ∕ Yamoscin (SEC)

No. = Identified peaks,

^aPositive ion mode,^

^bnegative ion mode.^

It was noticed that the first isolated active band (SEA) composed of two identified peaks (peaks no. 1 and 2) have the same molecular and fragment ions. Whereas, the mass spectrum of each peak indicated molecular ions at *m/z* 377 [M + Na]^+^, 355 [M + H]^+^, 707 [2 M − H]^−^ and 353 [M − H]^−^. Additionally, fragment ions at *m/z* 163 [M + H − 192]^+^ in the positive ion mode and *m/z* 191 [M − H − 162]^−^ in the positive mode which by the loss of quinic acid (192 amu) and caffeoyl (162) moieties, respectively. This was in agreement with that of chlorogenic acid^50,51^.

While the second active band (SEB) which refers to peak 3 showed [M + Na]^+^ at *m/z* 539, [M + H]^+^ at 517 and [M − H]^−^ at 515. Moreover, fragment patterns in + ve ion mode at *m/z* 355 [M + H − 162]^+^, 163 [M + H − 192]^+^, were consistent with the loss of caffeoyl (162 amu) and quinic (192) moieties, respectively as described previously. It also represented a fragment ion in the –ve mode at *m/z* 353 [M − H − 162]^−^ that further confirmed the neutral loss of caffeoyl (162) moiety. By comparing our MS results with the exploratory MS data in the mass bank as well as by reviewing the literature^50,52^, it can be suggested that this peak was identified as 3,5-dicaffeoyl quinic acid.

Finally, the active SEC band (peak 4) was characterized by molecular ion at *m/z* 869 [M + H]^+^, and upon fragmentation produced daughter ions at *m/z* 723 [M + H − 146]^+^, 576 [M + H − 146 − 146]^+^,414 [M + H − 146 − 146 − 162]^+^ due to three successive losses of sugar moieties [two of them were 146 amu for each deoxyhexose sugar (rhamnose), and one of 162 amu for hexose sugar]. Our findings matched with the literature^53^, which confirmed that this active compound was tentatively identified as a steroidal saponin (borassoside E or yamoscin). Thus, the fragmentation ions corroborated a loss of the first rhamnose moiety (146), accompanied by loss of the second rhamnose moiety (146), then a loss of glucose moiety (162) to give finally the steroidal aglycone at *m/z* 414.

## Discussions

Numerous biologically active metabolites are produced by the plant kingdom, and people have long used plant metabolic by-products^54^. The Solanaceae family of plants, which is home to many commercially and ecologically significant species, produces numerous substances that are classified as insecticides, acaricides, nematicides, fungicides, and bactericides^19^. Plants in the Solanaceae family have great potential to produce novel crop protection compounds^21^. Our current work aims to identify the active constituents of the promising *S. elaegnifolium* extract supported by a bioassay-guided approach and to determine the effective concentration on Grater wax moth larvae (*G. mellonella*) and Gram-negative bacteria *(E. carotovora*). DCM, EtOAc, and EtOH solvents were used successively to extract seeds and peel contents of *S*. *elaeagnifolium* fruits berries, while mucilage was extracted by EtOAc. The primary screening for antibacterial activity towards *E. carotovora* showed that seeds had the highest activity compared to other parts. Depending on the LC_50_ and LC_90_ values, the EtOH extract of seeds had the most promising mortality rates among other extracts. This finding was revealed in a study on the same plant by Hamouda et al*.*^55^, in which he found the insecticidal properties of the EtOH extract of *S. elaeagnifolium* seeds against *Spodoptera littoralis* revealed 100% larval mortality with the highest growth inhibition (59.68%) in comparison to leaves. The fruit extract of *S. melongena* and the leaf extract of *Capsicum annuum* showed potent antifeedant activity against *Spodoptera litura* and *Achaea janata* at different concentrations^56^. The insecticidal activity demonstrated by Jeyasankar et al*.*^57^ showed that *S*. *pseudocapsicum* seed extracts recorded maximum insecticidal activity against *Spodoptera litura* and *Helicover paarmigera* regardless of the concentration of used solvents.

The remarkable activities stated in the successive EtOH extract and its isolated active bands led us to identify these bioactive phytochemicals using LC-ESI-MS. Overall, LC-ESI-MS/MS of these active bands or fractions from the successive EtOH extract of *S. elaeagnifolium* seeds led to the tentative identification of three secondary metabolites, including two phenolic acids (chlorogenic acid and 3,5-dicaffeoyl quinic acid) and one steroidal saponin (borassoside E ∕ yamoscin).The two phenolic acids were previously identified from *S. elaeagnifolium*^13^. However, borassoside E or yamoscin had not previously been detected in our examined plant. Borassoside E was isolated from *S. violaceum* and had anticancer and anti-inflammatory activities. Yamoscin was identified from *S. violaceum* and *S. torvum* and had also anticancer and anti-inflammatory properties^52^. The chemical component of the first isolated band (SEA) that was annotated as chlorogenic acid showed the third capabilities on larval mortality and the second on bacterial inhibition. Chlorogenic acid is considered a botanical pesticide metabolite that occurs naturally in various plants since it has been involved in plant chemical defenses against insect herbivores^58^. For instance, it can be utilized as a thrips resistance factor in chrysanthemum^59^, and it is the principal compound in the anti-insect defenses of *Vernonia anthelmintica* Willd^60^. Besides, sweet potato weevil resistance is conferred via increased biosynthesis of chlorogenic acid in sweet^61^. In addition, several previous studies revealed that Chlorogenic acid is sufficient to control the armyworm *Mythimna separate*^62–64^. The second band (SEB) was suggested to be identified as 3,5-dicaffeoyl quinic acid, and showed only insecticide activity. This component has been shown to have insecticidal properties in some studies, including one performed by Poëssel et al*.*^65^, who reported that 3,5-dicaffeoyl quinic acid showed highly toxic effects against the larvae of *Myzus persicae* when mixed with an artificial diet. It was also demonstrated that dicaffeoyl quinic acids tend to have aphicidal activity against *Acyrthosiphon pisum*^66,67^. 3,5-dicaffeoyl quinic acid isolated from *Chrysanthemum morifolium* showed phytotoxic and insect growth regulating activity^68^. This compound reduced both growth and photosynthesis of *Lemna gibba* L. and enhanced or reduced growth of the cabbage looper (*Trichoplusia ni*) and gypsy moth (*Lymantria dispar* L.). Moreover, the herbicidal activity of previously isolated constituents from *S. elaeagnifolium* Seeds by chlorogenic acid highly decreased *Portulaca oleracea* total biomass fresh weight by (86.5%). However, the lowest activity (63.6%) was achieved by 3,5-dicaffeoyl quinic acid compared with the control^13^. The third active band (SEC), shows maximum larval mortality and antibacterial activity and is tentatively annotated as Borassoside E ∕ yamoscin. A similar study was carried out by Dolma et al.^69^, that the compounds of Brassoside E caused a mortality rate of 74% against *Aphis craccivora* after 96 h of treatment at an LC_50_ value of 3467.1 mg L^−1^.

In conclusion, this study considered that the EtOH seed fraction of *S. elaeagnifolium* which extracted successively after DCM and EtOAc fractions can be potentially used for the control of *G. mellonella* and *E. carotovora*. These activities are based on the isolated bioactive phytochemicals [steroidal saponin (SEC) > dicaffeoylquinic acid (SEB) > chlorogenic acid (SEA)] that are effective against *G. mellonella*. While the first and third compounds can be utilized in bacteria suppression of *E. carotovora*. These findings emphasize the significance of invasive *S. elaeagnifolium* weeds as a promising source of natural pesticides and the value addition of weeds in the future. Finally, the research of a suitable formulation is required for delivering the active substances to the target pests to aid the use of natural pesticides in agro-technological treatments and quality control against pests and involved in integrated pest management.

### Supplementary Information

Supplementary Information.

## Data Availability

All data generated or analysed during this study are included in this published article and its Supplementary Information file.
