# Supplementary material for: Bioactivity of silverleaf nightshade (Solanum elaeagnifolium Cav.) berries parts against Galleria mellonella and Erwinia carotovora and LC-MS chemical profile of its potential extract
Source: Sci Rep. 2024 Aug 13;14:18747. doi: 10.1038/s41598-024-68961-z (PMC11322330; doi:10.1038/s41598-024-68961-z)
Supplement: Supplementary file 1 — Supplementary Information. [file 41598_2024_68961_MOESM1_ESM.docx]

**Supplementary material**

**Bioactivity of Silverleaf nightshade (*Solanum elaeagnifolium* Cav.) berries parts against *Galleria mellonella* and *Erwinia carotovora* and LC-MS chemical profile of its potential extract**

**Abdel Nasser A. Kobisi^1^, Mohamed A. Balah^1*^, Ahmed R. Hassan^2^**

^1^Plants Protection Department, Desert Research Center, Cairo, Egypt

^2^Medicinal and Aromatic Plants Department, Desert Research Center, Cairo, Egypt

***Corresponding author: **Mohamed A. Balah**

*E-mail address*: [mbaziz1974@gmail.com](mailto:mbaziz1974@gmail.com)

**Bioactivity of certain fruit berries parts of Silverleaf nightshade (*Solanum elaeagnifolium* Cav.) against *Galleria mellonella* and *Erwinia carotovora* and LC-MS chemical profile of its potential extract**

**Abstract**

Natural products received much attention as an environmentally beneficial solution for pest management. Therefore, the extracts of invasive Silverleaf nightshade (*Solanum elaeagnifolium* Cav.) weeds using their berries parts (seeds, peels and mucilage) supported by bioassay-guided fractionation were tested against both the greater wax moth (*Galleria mellonella*) and potatoes erwinia (*Erwinia carotovora*)*.* The seeds and peels of *S. elaeagnifolium* were successively extracted by maceration using dichloromethane (DCM), ethyl acetate (EtOAc), and ethanol (EtOH), respectively. While, EtOAc extracted its Mucilage. The successive EtOH extract of the plant seeds had promising inhibition efficacy and the best minimal inhibition concentration (MIC) of 50 µg/ml against *E. Carotovora* amongst other extracts (DCM & EtOAc extracts of the plant berries parts). Depending on dose response activity, EtOH extract had *G. mellonella* larval mortality and pupal duration rates (LC_50_; 198.30 and LC_95_; 1294.73 µg/ml), respectively. Additionally, this ethanol extract of seeds was fractionated using preparative TLC to three characteristic bands. The insecticidal and bacterial activities of these isolated bands (SEA, SEB, and SEC) were evaluated at a dose of 100 µg/ml, causing mortality by 48.48, 62.63 and 92.93% (*G. mellonella* larvae) and inhibition by 15.22, 0.00 and 31.66 mm (*E. carotovora*), respectively. Moreover, the separated major three bands were tentatively identified using LC-ESI-MS analysis revealing the presence of two phenolic acids; chlorogenic acid (SEA) and dicaffeoyl quinic acid (SEB) in addition to one steroidal saponin (SEC) annotated as borassoside E or yamoscin. Finally, the plant seeds' successive EtOH extract as well as its active constituents, exhibited potential broad-spectrum activity and the ability to participate in future pest management initiatives. A field study is also recommended to validate its bio-efficacy against selected pests and to develop its formulations.

**Keywords:** *Solanum elaeagnifolium*, *Galleria mellonella*, *Erwinia carotovora*, insecticidal activity, bioassay guided approach, LC-MS.

**
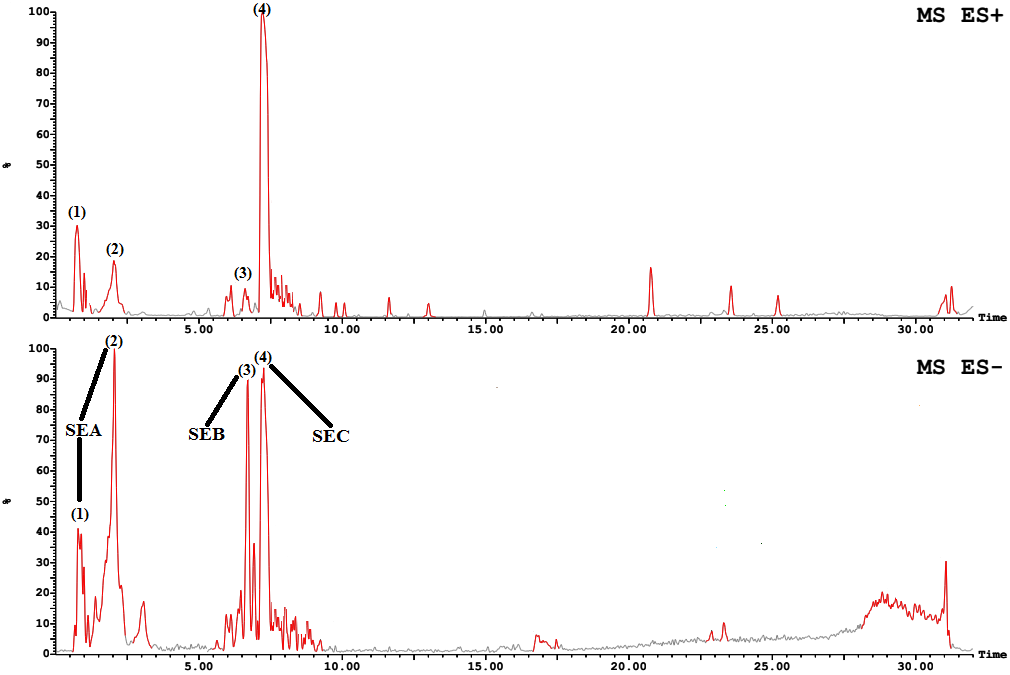
**

**Fig. S1:** LC-ESI-MS base peak chromatogram of *S. elaeagnifolium* ethanol seed extract and its active fractionated bands.


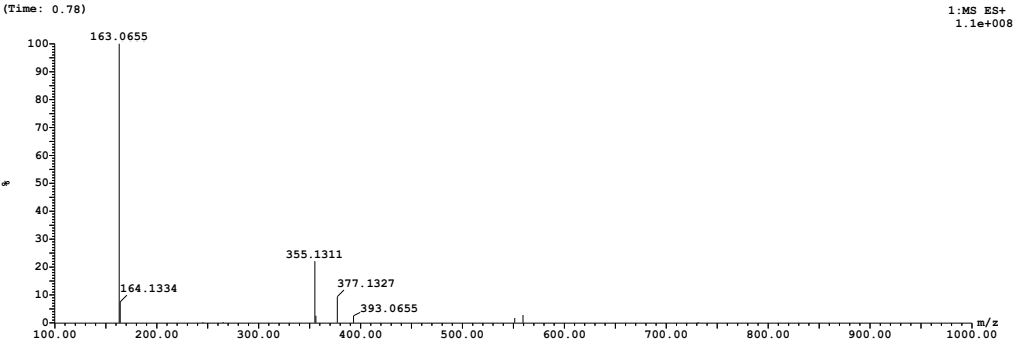


**Fig. S2:** ESI-MS of peak 1 (**SEA**) in the positive ion mode


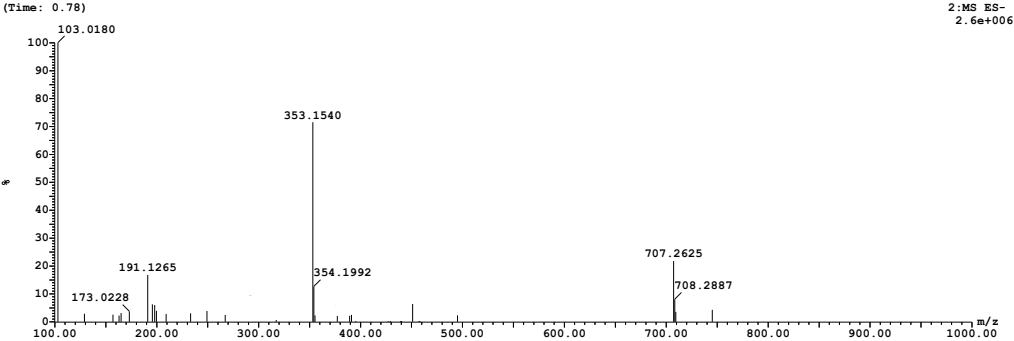


**Fig. S3.** ESI-MS of peak 1 (**SEA**) in the negative ion mode


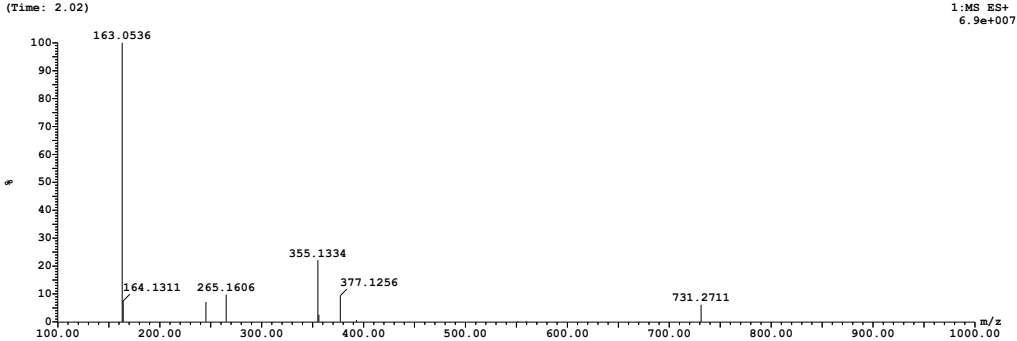


**Fig. S4.** ESI-MS of peak 2 (**SEA**) in the positive ion mode


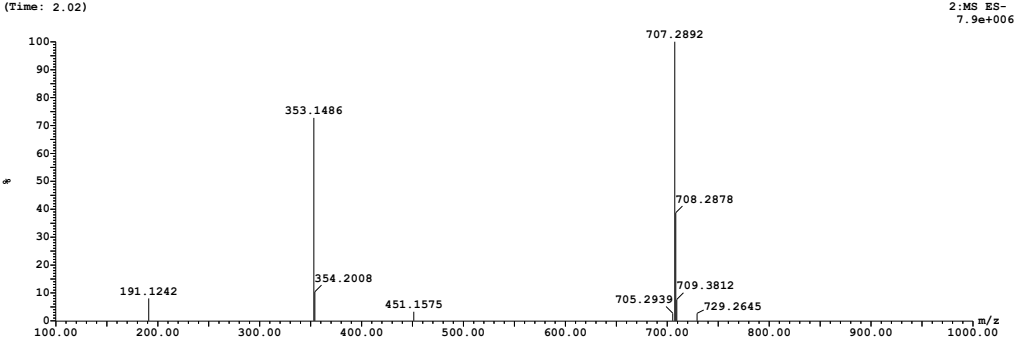


**Fig. S5.** ESI-MS of peak 2 (**SEA**) in the negative ion mode


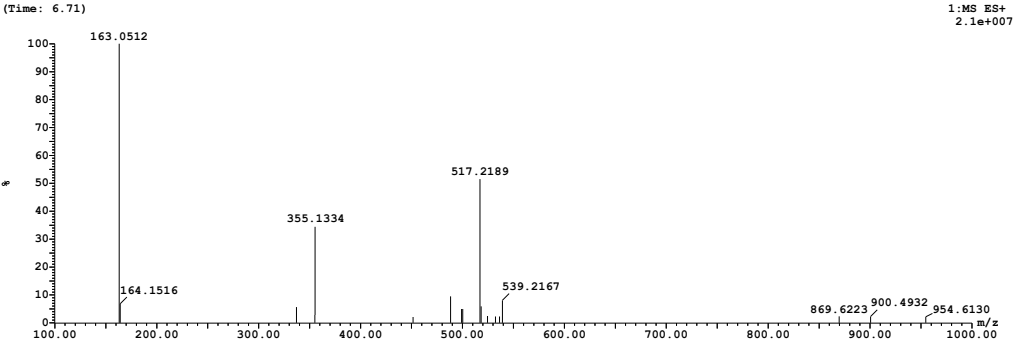


**Fig. S6.** ESI-MS of peak 3 (**SEB**) in the positive ion mode


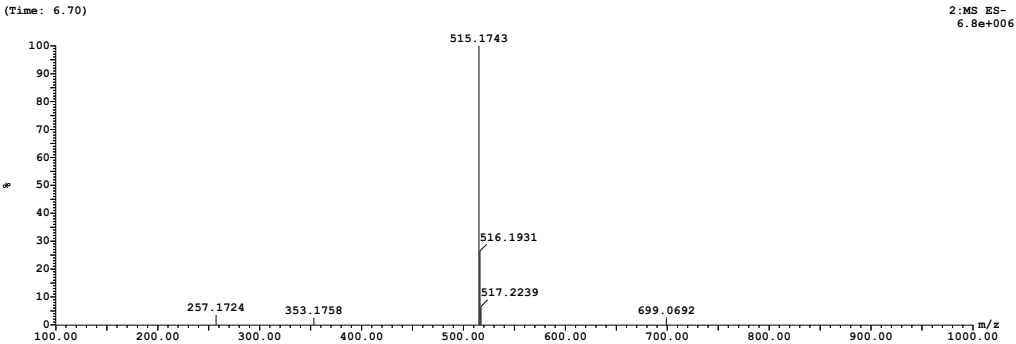


**Fig. S7.** ESI-MS of peak 3 (**SEB**) in the negative ion mode


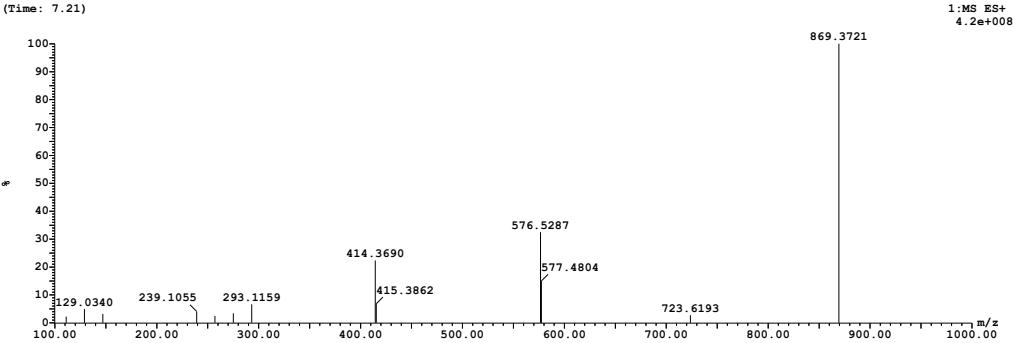


**Fig. S8.** ESI-MS of peak 4 (**SEC**) in the positive ion mode


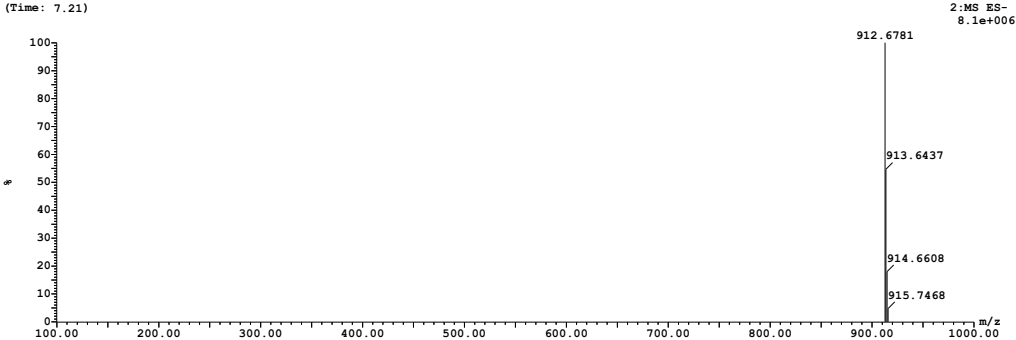


**Fig. S9.** ESI-MS of peak 4 (**SEC**) in the negative ion mode


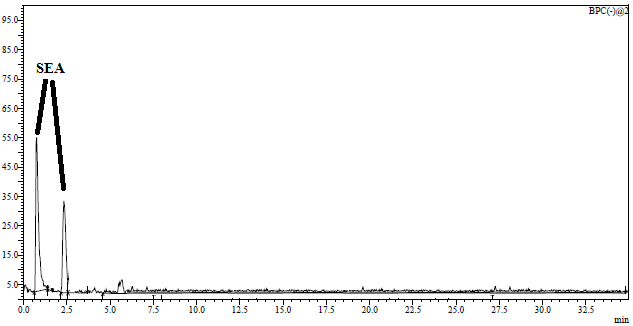


**Fig. S10:** LC-ESI-MS base peak chromatogram of SEA fractionated band in the negative ion mode


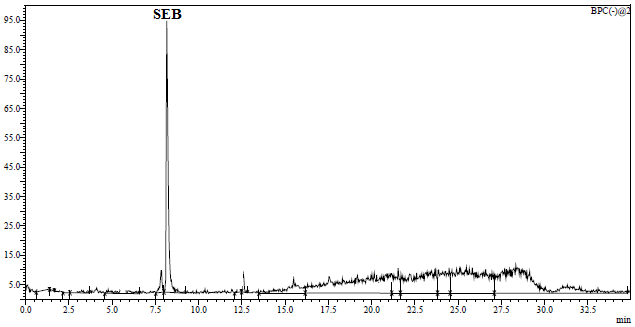


**Fig. S11:** LC-ESI-MS base peak chromatogram of SEB fractionated band in the negative ion mode


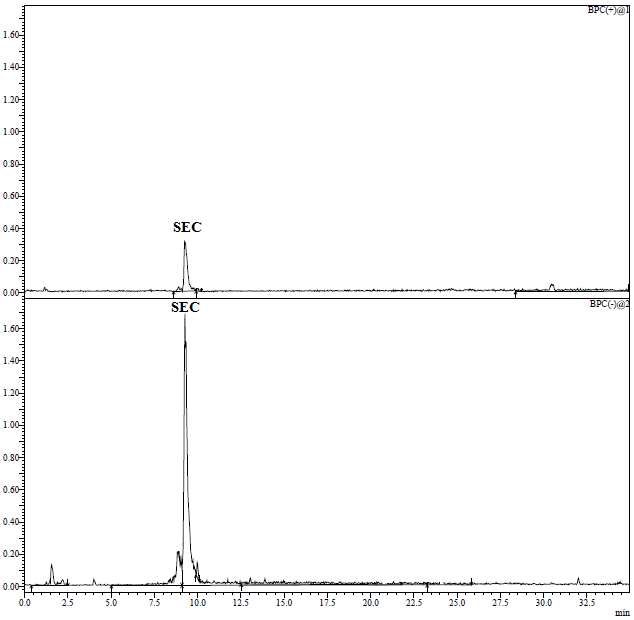


**Fig. S12:** LC-ESI-MS base peak chromatogram of SEA fractionated band in both positive and negative ion modes
